# A novel deep learning framework for automatic scoring of PD-L1 expression in non-small cell lung cancer

**DOI:** 10.17305/bb.2025.12056

**Published:** 2025-03-03

**Authors:** Saidul Kabir, Muhammad E H Chowdhury, Rusab Sarmun, Semir Vranić, Rafif Mahmood Al Saady, Inga Rose, Zoran Gatalica

**Affiliations:** 1 Department of Electrical and Electronic Engineering, University of Dhaka, Dhaka, Bangladesh; 2 Department of Electrical Engineering, Qatar University, Doha, Qatar; 3 College of Medicine, QU Health, Qatar University, Doha, Qatar; 4 Reference Medicine, Phoenix, Arizona, United States of America

**Keywords:** Programmed death-ligand 1, PD-L1, non-small cell lung cancer, NSCLC, artificial intelligence, AI, deep learning, classification, segmentation

## Abstract

A critical predictive marker for anti-PD-1/PD-L1 therapy is programmed death-ligand 1 (PD-L1) expression, assessed by immunohistochemistry (IHC). This paper explores a novel automated framework using deep learning to accurately evaluate PD-L1 expression from whole slide images (WSIs) of non-small cell lung cancer (NSCLC), aiming to improve the precision and consistency of tumor proportion score (TPS) evaluation, which is essential for determining patient eligibility for immunotherapy. Automating TPS evaluation can enhance accuracy and consistency while reducing pathologists’ workload. The proposed automated framework encompasses three stages: identifying tumor patches, segmenting tumor areas, and detecting cell nuclei within these areas, followed by estimating the TPS based on the ratio of positively stained to total viable tumor cells. This study utilized a Reference Medicine (Phoenix, Arizona) dataset containing 66 NSCLC tissue samples, adopting a hybrid human–machine approach for annotating extensive WSIs. Patches of size 1000 × 1000 pixels were generated to train classification models, such as EfficientNet, Inception, and Vision Transformer models. Additionally, segmentation performance was evaluated across various UNet and DeepLabV3 architectures, and the pre-trained StarDist model was employed for nuclei detection, replacing traditional watershed techniques. PD-L1 expression was categorized into three levels based on TPS: negative expression (TPS < 1%), low expression (TPS 1%–49%), and high expression (TPS ≥ 50%). The Vision Transformer-based model excelled in classification, achieving an F1-score of 97.54%, while the modified DeepLabV3+ model led in segmentation, attaining a Dice Similarity Coefficient of 83.47%. The TPS predicted by the framework closely correlated with the pathologist’s TPS at 0.9635, and the framework’s three-level classification F1-score was 93.89%. The proposed deep learning framework for automatically evaluating the TPS of PD-L1 expression in NSCLC demonstrated promising performance. This framework presents a potential tool that could produce clinically significant results more efficiently and cost-effectively.

## Introduction

Lung cancer, with nearly two million new cases each year, is the most prevalent cancer globally [1]. Patients with stage IV non-small cell lung cancer (NSCLC) have a survival rate of only 5% [2, 3].

The PD-1 receptor and its ligands, PD-L1 and PD-L2, belong to a family of immune checkpoint proteins. These molecules function as co-inhibitory factors for T cells, effectively dampening immune responses. The interaction between PD-1 and PD-L1 plays a crucial role in regulating the timing of immune system activation [4]. Expression of PD-L1 on tumor cells (TCs) allows them to bind to PD-1 receptors on activated T cells, enabling TCs to evade anticancer immunity [5, 6]. Monoclonal antibodies that block this interaction between PD-1 and PD-L1 can restore the immune system’s ability to recognize and destroy cancer cells [7, 8].

Researchers have developed several inhibitors based on this mechanism of action. Existing ICIs, including anti-PD-1 and anti-PD-L1 inhibitors, have shown promising results in clinical trials [9, 10]. Immune checkpoint inhibitors (ICIs) targeting the programmed cell death-1 (PD-1)/programmed death-ligand 1 (PD-L1) pathway have significantly improved survival rates for patients diagnosed with NSCLC [11–13].

The Food and Drug Administration (FDA) has approved DAKO PD-L1 22C3 PharmDx as a companion diagnostic test for the immunotherapeutic drug pembrolizumab in patients with NSCLC [14]. Evaluating PD-L1 expression is crucial in managing patients, as it helps identify those who are more likely to respond to pembrolizumab. This applies to its use as a first- or second-line monotherapy or alongside standard chemotherapy [15].

The tumor proportion score (TPS) is calculated as the percentage of TCs showing at least partial membranous staining for PD-L1, relative to the total number of TCs [16]. This calculation excludes tumor-associated immune cells (ICs), normal, necrotic, and non-neoplastic cells. The TPS can be defined using the formula 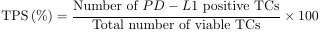.

Pathologists usually estimate TPS through microscopic examination. For specimens with heterogeneous tumor regions exhibiting varying PD-L1 expression, TPS is determined by calculating the average percentage of stained TCs across multiple divided tumor regions. This approach accounts for the spatial heterogeneity of PD-L1 expression often encountered in tumor samples, providing a more representative assessment of overall PD-L1 status [17].

PD-L1 expression is commonly observed in NSCLC [18–21] and is predictive of response to ICI. However, scoring PD-L1 expression in NSCLC specimens presents significant challenges, particularly in advanced-stage patients [22, 23]. This process requires experienced evaluation to ensure accurate tumor classification. Inter-observer variability among pathologists during manual scoring has been reported (kappa score as low as 0.45), potentially leading to inconsistent results. Pathologist-dependent scoring introduces an inherent source of error, as noted in multiple studies, which becomes particularly pronounced in cases of low PD-L1 expression [24, 25]. Moreover, manually evaluating PD-L1 expression can be a tedious process susceptible to subjectivity [16]. This subjectivity arises from the difficulties associated with accurately quantifying cellular elements across entire slide sections. The process is further complicated by the subjective nature of stain intensity assessment, introducing additional variability. These factors collectively contribute to challenges in maintaining reproducibility and inter-observer consistency in PD-L1 scoring. The complexity of this assessment underscores the need for potentially automated solutions to enhance the accuracy and reproducibility of PD-L1 expression evaluation in NSCLC specimens.

Deep learning (DL) has been widely integrated into the healthcare sector in recent years, demonstrating its potential to address diagnostic inconsistencies. By leveraging deep learning techniques, medicine can benefit from these models’ ability to identify complex patterns and features within extensive datasets, leading to precise and consistent evaluations [26–28]. This technological advancement can mitigate reliance on individual medical practitioners and reduce variability in subjective interpretations among different observers. The extensive application of deep learning in healthcare underscores its transformative impact on medical diagnostics and treatment [29].

Digital image analysis techniques offer a promising approach to addressing the limitations in PD-L1 scoring, especially in the scoring of whole tissue sections. Artificial intelligence (AI) methodologies, particularly those employing deep learning,algorithms, have demonstrated the potential to augment pathologists’ capabilities, enhancing diagnostic accuracy, inter-observer concordance, and overall efficiency [30–34].

Previous investigations have primarily focused on evaluating the correlation between pathologist-derived and automated PD-L1 scores. Findings from these studies indicate that automated systems demonstrate accuracy comparable to that of experienced pathologists in PD-L1 expression assessment [35–40]

In the studies by Taylor et al. [38] and Sha et al. [41], PD-L1 TPS was calculated at the field-of-view level by measuring tumor region areas. However, this regional area ratio-based method lacks precision, as it does not align with clinical guidelines, which mandate that TPS be determined based on TC counts. Methods that calculate at the cellular level have demonstrated superior results, as TPS calculations are derived from individual TCs [42]. Subsequent research has shown that the open-source program QuPath, used for scoring PD-L1 in NSCLC, has produced promising results [43, 44]. Notably, most studies have utilized watershed-based image processing techniques to identify cell nuclei [36, 39]. While this approach may be effective in clear-cut cases, it tends to struggle in challenging scenarios involving variations in stain intensity and coloring.

Huang et al. [45] tested an AI-assisted scoring system for assessing PD-L1 expression in NSCLC using the UNet segmentation model, which was trained, validated, and tested on whole slide images (WSIs). The results showed that the model’s output correlated strongly with the gold standard TPS and performed comparably to experienced pathologists, though it was less effective in high TPS groups due to false positives. Nevertheless, it demonstrated potential for aiding routine diagnosis by pathologists. This study employed a basic UNet architecture to segment positive and negative nuclei. However, this straightforward approach may lead to inaccuracies in more complex cases involving color intensity. Wu et al. [39] proposed developing an AI-based system using WSIs from the 22C3 assay, incorporating a UNet architecture with residual blocks to segment tumor areas and automatically calculate the TPS of PD-L1 expression. The system showed strong consistency with trained pathologists and improved the efficiency and workload of untrained pathologists, demonstrating high precision in both the 22C3 and SP263 assays. Cheng et al. [46] developed a YOLO-based AI model for assessing PD-L1 expression in lung cancer patients, including 1288 participants. The model used a detection algorithm to identify positive and negative nuclei in TCs. Its diagnostic results were consistent with those of pathologists, demonstrating similar performance across different lung cancer subtypes and suggesting that AI-assisted diagnostic methods are promising tools for enhancing clinical pathologist efficiency.

Liu et al. [36] developed a novel Automated Tumor Proportion Scoring System (ATPSS) to compare image analysis results with pathologist scores. The ATPSS employs a three-stage process that integrates ResNet-UNet-based architectures for detecting tumor regions and nuclei, alongside image processing techniques to identify positive staining. The ATPSS demonstrated a high correlation with pathologist scores, achieving a mean absolute error (MAE) of 8.65 and a Pearson correlation coefficient (PCC) of 0.9436. However, image-processing-based detection of positive regions may erroneously classify artifacts and stained ICs as positive cancer cells.

Ito et al. [47] developed a model to calculate the TPS of the PD-L1 22C3 assay and evaluate its effectiveness in assisting pathologists. They used a UNet architecture to segment nuclei and a DeepLab architecture to segment tumor areas. The findings highlight the AI-assisted system’s potential to enhance pathologists’ accuracy, particularly in challenging cases where their judgments were inconsistent.

In this study, a comparative analysis of the segmentation performance of various UNet architectures with different encoders was conducted alongside a modified DeepLabV3+ architecture. Additionally, we developed an end-to-end framework for calculating TPS from WSIs, incorporating a classification stage to enhance resilience against artifacts and misidentified positive ICs. For nuclei detection, we employed a deep learning-based approach complemented by image processing techniques, addressing the limitations of the commonly used watershed method, which often fails in cases of overstaining and low intensity.

The major contributions of this study are as follows.

- Development of a novel end-to-end framework for the automated assessment of PD-L1 expression TPS using WSIs from surgical resections.
- Annotation of entire surgical resection WSIs through a combined human–machine approach.
- Comparative analysis of various segmentation networks, including UNet with different encoders and a modified DeepLabV3+ architecture, for tumor region segmentation.
- Deep learning-based cancer cell nuclei detection ensures robust and precise TPS calculation.

## Materials and methods

### Dataset

This study examined 66 surgically obtained tissue samples from patients with confirmed NSCLC. The specimens were collected at Reference Medicine (Phoenix, AZ, USA) between January 2020 and October 2022. For each case in the dataset, PD-L1 immunohistochemistry (IHC) slides were prepared using the Dako Autostainer Link 48 platform, following the automated staining protocol with the PD-L1 22C3 antibody. TPS was used for PD-L1 assessment following the PD-L1 IHC 22C3 pharmDx Interpretation Manual NSCLC [17]. All slides were digitized using the Motic EasyScan Pro slide scanner. Table 1 presents the baseline characteristics of the NSCLC cohort.

**Table 1:** Baseline characteristics of the NSCLC patient cohort

**Characteristic**	**Dataset (*N* ═ 66)**	
Age, years	Mean Range	66 50–82
Sex	Men Women	36 (54.5%) 30 (45.5%)
Specimen site	Primary (lung) Metastatic (lymph nodes)	64 (97%) 2 (3%)
Tumor type	Adenocarcinoma Squamous cell carcinoma Other subtypes of NSCLC	46 (69.7%) 13 (19.7%) 7 (10.6%)
TPS	<1% 1%–49% ≥50%	30 (45.5%) 22 (33.3%) 14 (21.2%)

Abbreviations: TPS: Tumor proportion score; NSCLC: Non-small cell lung cancer.

### Data processing

**Figure 1.**
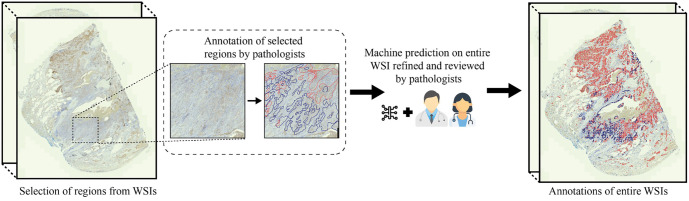
**Annotation procedure based on human–machine collaborative approach.** Abbreviation: WSI: Whole slide image.

In this study, we employed a hybrid machine–human approach for case annotation. From each WSI, a representative tumor area was selected for manual annotation. This approach was adopted to mitigate the considerable challenges associated with annotating entire WSIs, including annotator fatigue and the time-intensive nature of the task. Two independent pathologists conducted annotations on the selected regions from each WSI using QuPath software (Version 0.2.2) [43]. The annotation process involved categorizing the tissue into three distinct classes: class 0 (non-TC regions), class 1 (TC regions with PD-L1 expression), and class 2 (TC regions without PD-L1 expression). A segmentation model was initially trained on these manually annotated tumor regions obtained from the WSIs. Once trained, the model was utilized to predict tumor regions throughout the entire WSIs. This preliminary machine-generated segmentation provided a basis for the initial annotation of tumor areas, thereby streamlining the subsequent review and annotation process, allowing for the annotation of large WSIs with ease. The two pathologists then conducted a comprehensive review of the automated annotations to adjust and refine them. This process enhanced the reliability of the training data for further segmentation model training. This iterative refinement was crucial for training robust models capable of precise tumor segmentation in the WSIs, thereby supporting more effective and efficient pathological assessments. Figure 1 visualizes the annotation approach undertaken in this study.

**Figure 2.**
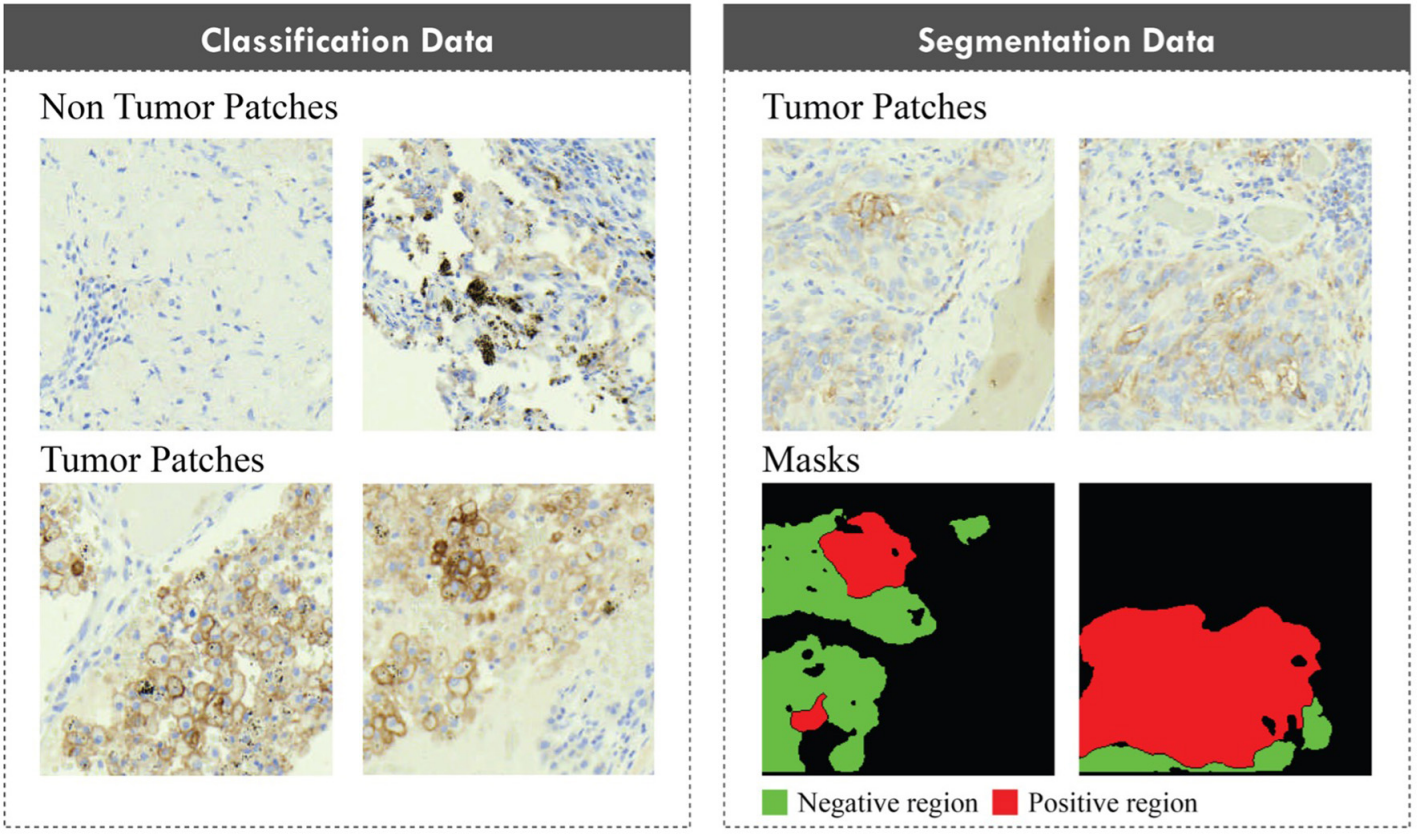
Example of patches in classification and segmentation dataset.

Patches of size 1000 × 1000 pixels were generated from the WSIs at the highest magnification (40× optical magnification, 0.267 µm/pixel). The WSIs were divided into five folds for cross-validation, with the patches from each image assigned to their respective fold. Two distinct datasets were created from the extracted image patches. The first dataset was designed to facilitate the classification of each patch based on the presence of tumor tissue, while the second was intended for segmenting regions into positive and negative tumor areas. For the classification dataset, patches devoid of TCs were labeled as “non-tumor” whereas patches containing any TCs were labeled as “tumor.” For the segmentation dataset, both the patches and their corresponding masks were generated based on annotations provided by pathologists. Figure 2 shows an example of a classification and segmentation dataset.

### Proposed method

**Figure 3.**
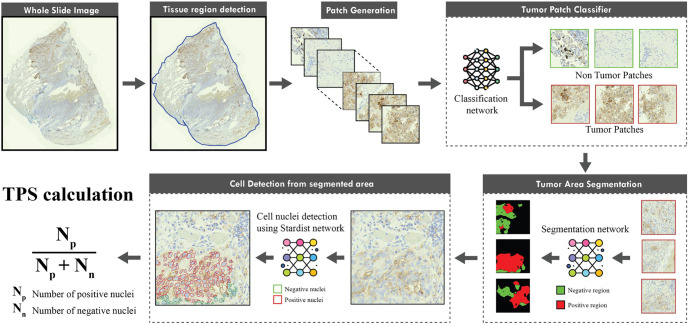
**A proposed automated framework for TPS calculation of PD-L1 expression.** Abbreviations: TPS: Tumor proportion score; PD-L1: Programmed death-ligand 1.

We propose a novel end-to-end automated framework for determining TPS in DAB-stained NSCLC WSI. It consists of three key stages: tumor patch classification, tumor area segmentation, and nuclei detection. Initially, a deep learning model identifies and excludes non-tumor patches by discarding patch images containing artifacts or lacking TCs. In the subsequent stage, segmentation networks predict pixel-wise classifications (negative or positive) within the tumor patches, delineating the negative and positive regions. Finally, we employ a secondary neural network that utilizes the pre-trained “StarDist” model to facilitate cell detection within the annotated tumor regions [48]. Figure 3 illustrates the flow of the proposed framework.

### Tumor patch classification

The first stage for automated tumor patch identification in WSIs of tumor regions was developed using convolutional neural network (CNN)-based classification models. CNN-based architectures are highly popular in image classification due to their ability to capture spatial patterns in local regions and learn abstract features at multiple levels. Their hierarchical structure and shared weights have enabled them to achieve state-of-the-art results on standard classification datasets and benchmarks [29, 49]. Vision Transformer models leverage self-attention mechanisms, which can capture global dependencies and interactions over long distances, effectively representing contexts at both local and global scales and surpassing the limitations of traditional CNN architectures in image classification. In image classification tasks, pre-trained models initialized with ImageNet weights undergo fine-tuning on smaller, task-specific datasets. This fine-tuning process involves replacing the model’s final layers and adjusting the weights at a lower learning rate, thereby significantly reducing training time and costs by utilizing previously learned generic features.

In this study, we conducted an extensive evaluation of various deep-learning models for classification tasks. The performance of the top three performing models—Inception v3 [50], EfficientNet [51], and a Vision Transformer-based model [52]—was reported based on their classification performance.

EfficientNet-B0 [51], the foundational model in the EfficientNet series, employs compound scaling to enhance CNNs by adjusting their width, depth, and resolution. Developed via neural architecture search, this method optimizes both accuracy and computational efficiency by uniformly scaling network dimensions with fixed coefficients, ensuring balanced growth and effectiveness.

Inception v3 [50], developed by Google, is an advanced CNN that improves on its predecessors by employing factorized convolutions and expanded inception modules to reduce parameters without sacrificing depth or width. It also introduces label smoothing to prevent overfitting, enhancing its performance in complex image classification tasks. This architecture optimally balances computational efficiency with robust capabilities, making it highly effective for various image-processing applications.

The Vision Transformer (ViT) [52] adapts the transformer architecture, originally designed for natural language processing, to image classification tasks. ViT splits the input image into fixed-size patches, transforms them into token embeddings with added positional embeddings for spatial context, and processes these through several transformer encoder layers. These layers equipped with self-attention mechanisms, allow the model to capture complex relationships across the image, and feed-forward networks that apply nonlinear transformations to the data. The token embeddings are then processed by a classifier head.

### Tumor area segmentation

This stage of the framework identifies negative and positive regions in the patches using a segmentation network trained on pathologist annotations. The UNet architecture [53], developed for precise image segmentation in the biological domain, features a U-shaped design with an encoder that compresses and a decoder that decompresses. The encoder consists of convolutional layers with ReLU activation and max pooling, which reduce spatial dimensions while increasing feature depth. The decoder then restores the feature maps to their original spatial dimensions. UNet’s skip connections link encoder and decoder layers, merging high-level and detailed information in the output. A common approach to improving the architecture’s performance involves integrating advanced encoder architectures. Specifically, the UNet framework for image segmentation is enhanced by incorporating encoders such as DenseNet [54], which utilizes dense connections, and EfficientNet [51], known for its optimized performance in resource-limited settings. These enhancements facilitate feature reuse, alleviate the issue of vanishing gradients, and promote feature propagation, thereby achieving more precise segmentation outcomes. This study presents comparative results between the conventional U-Net and the modified U-Net frameworks employing DenseNet and EfficientNet encoders. DeepLabV3 [55] and DeepLabV3+ [56] are advanced models designed for semantic segmentation, aiming to enhance object segmentation at various scales and achieve more precise boundaries. These models are significant enhancements of the DeepLab series, leveraging deep CNNs for high-resolution image segmentation. DeepLabV3, introduced by Chen et al., integrates an atrous convolution technique to expand filter ranges and capture context at multiple scales without losing resolution. It features an atrous spatial pyramid pooling (ASPP) module that analyzes a convolutional feature layer using filters with varying sampling rates and effective fields of view, effectively capturing objects and context at various scales.

**Figure 4.**
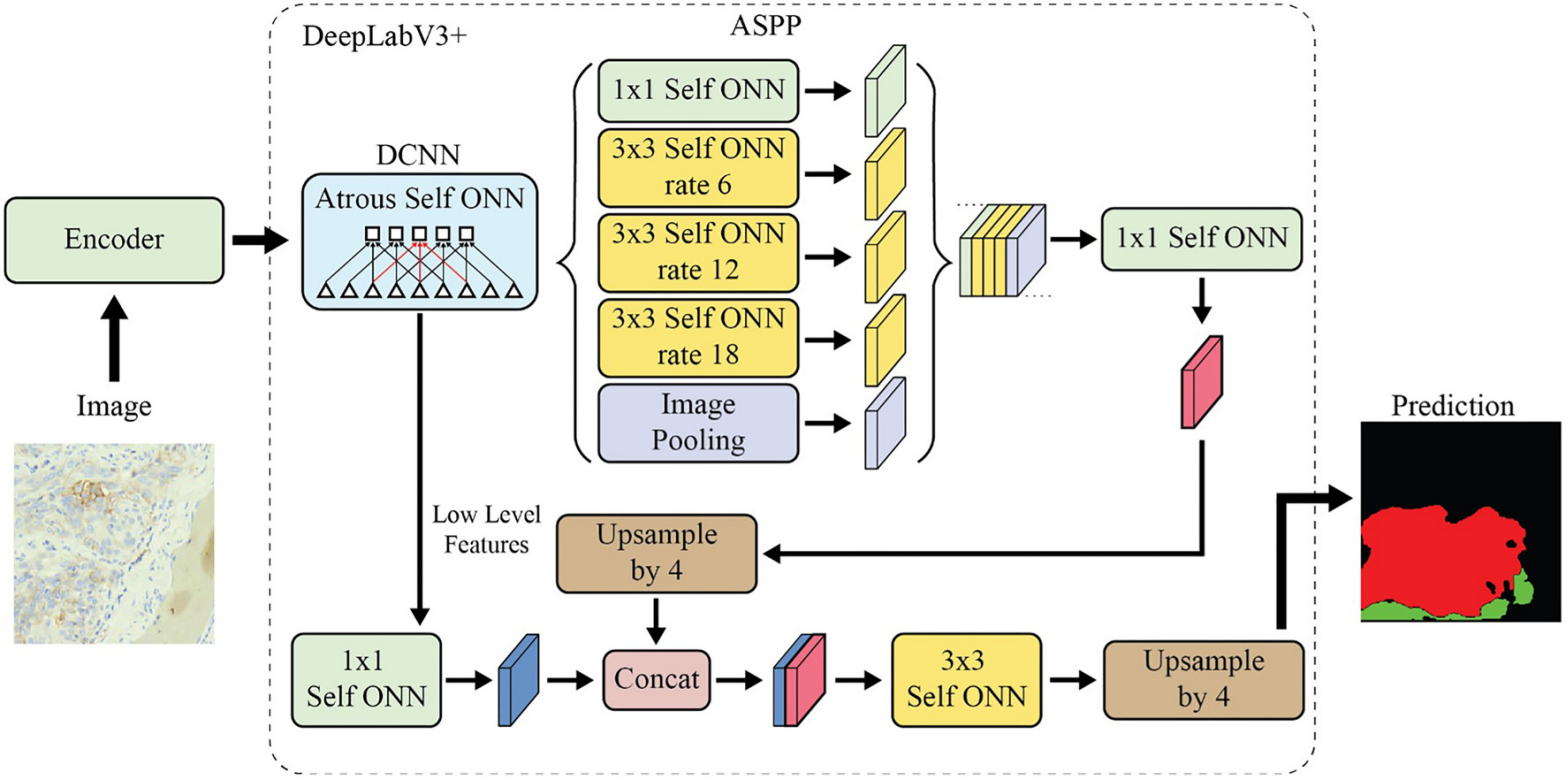
**Architecture of modified DeepLabV3+ network.** Abbreviations: ONN: Operational neural network; ASPP: Atrous spatial pyramid pooling.

We enhanced the DeepLabV3+ network by incorporating Self-Organized Operational Neural Networks (Self-ONN) [57], which have been shown to outperform traditional CNNs. CNNs, with their homogeneous, linear structures, do not fully replicate the complexity of biological neural systems. Addressing these limitations, Generalized Operational Perceptrons (GOPs) and Operational Neural Networks (ONNs) introduce heterogeneous and non-linear architectures. GOPs, drawing inspiration from biological mechanisms, are adept at handling complex tasks where traditional models falter. ONNs extend these advancements by incorporating a variety of operational units per neuron, such as nodal and pool operators, which transcend standard linear convolutions. This approach retains fundamental CNN principles like weight sharing and localized connectivity while expanding the functional capabilities of the network layers. In our modified architecture, all CNN layers in the DeepLabV3+ network were replaced with Self-ONN layers. Figure 4 presents the architecture of the Self-ONN-based DeepLabV3+ model. Additionally, we conducted comparative analyses between the original and modified networks to highlight the improved performance of our Self-ONN-based architecture.

### Nuclei detection and TPS calculation

The objective of this stage of the framework was to accurately identify the nuclei within both positive and negative tumor regions. For this purpose, we employed a pre-trained network, StarDist [48], a deep learning-based method designed for object detection and segmentation in biological images. It distinguishes itself from conventional object detection techniques by employing star-convex polygons for object representation, as opposed to the traditional use of axis-aligned bounding boxes. This technique involves regressing distances from each pixel within an object to its boundary along a set of predefined radial directions. These distance calculations are relevant only for pixels that have been definitively identified as parts of an object, where object probabilities are determined through a predictive model.

To further refine object representations, non-maximum suppression is utilized to select the polygons that most accurately represent objects based on the highest computed object probabilities. These probabilities are determined by the normalized Euclidean distances to the nearest background pixel, focusing on polygons nearer to the object’s center for more accurate depictions. The framework employs the UNet architecture, augmented with an additional convolutional layer designed to enhance feature discrimination before the output phases. Object probabilities are derived from a sigmoid-activated convolutional layer, whereas polygon distances are produced directly, scaled by the number of radial directions without subsequent activation. Collectively, this approach offers a refined and computationally efficient alternative to traditional object detection methods, significantly improving the accuracy of complex image segmentation, especially in medical imaging scenarios where precise object delineation is crucial.

**Figure 5.**
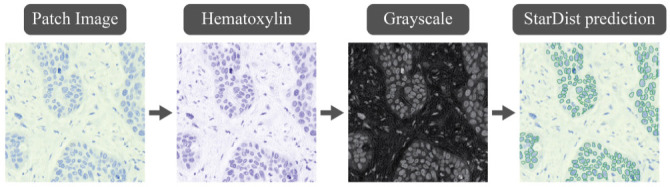
Detection of cell nuclei using the StarDist network.

This innovative approach facilitates precise and adaptable modeling of the typically irregular and complex shapes observed in biological microscopy images. A pre-trained model was used in this work, which required some image preprocessing. Initially, the patch image was deconvoluted to separate stain channels, specifically to isolate the hematoxylin channel. This channel was then converted to grayscale, and a blurring filter was applied to reduce noise. Subsequently, the StarDist model was utilized to detect nuclei within both the positive and negative tumor regions. The procedure is demonstrated in Figure 5. Any cell detected within the region identified as positive during the segmentation stage was classified as a PD-L1-positive cancer cell. Conversely, any nucleus located within the negative region was classified as negative. Following this, the TPS was calculated as the ratio of the total number of stained positive TCs to the total number of viable TCs.

### Training and testing methodology

A five-fold cross-validation approach was utilized to ensure a comprehensive and accurate evaluation of the deep learning models’ performance, dividing the 66 WSIs into five folds. In this approach, performance evaluation is conducted on the entire dataset, with each fold used as the test set once, while the remaining folds are used for training and validation. This fold split was consistently applied to both classification and segmentation training. From the WSI, patches measuring 1000 by 1000 pixels at 40× magnification were created, resulting in a total of 120,360 patches.

Classification and segmentation networks were trained separately on the patches. Training for both classification and segmentation was conducted over 100 epochs, with the best epoch’s result saved based on validation set performance. To prepare the input for the models, all patch images were resized to 224 × 224 pixels for classification and 256 × 256 for segmentation, as these are the image dimensions required for using the ImageNet weights. Preliminary training involved experimenting with various learning rates, ultimately selecting 0.0001 with the Adam optimizer for its optimal results.

All experiments were conducted on a hardware setup consisting of an NVIDIA GeForce RTX 3090 with 32 GB of GPU memory, a 36-core Intel Xeon(R) CPU @ 2.30 GHz, 64 GB of system memory, Python 3.9.16, and PyTorch version 1.13.

## Results

### Performance metrics

The performance of classification and segmentation tasks was evaluated using a comprehensive set of metrics. For the classification task, we utilized precision, recall, F1-score, and accuracy as the primary evaluation metrics. In the segmentation task, the assessment was conducted using Intersection over Union (IoU), Dice Similarity Coefficient (DSC), True Positive Rate (TPR), False Positive Rate (FPR), and specificity. The mathematical formulations of these metrics are detailed below: 
(1)
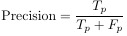

(2)
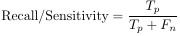

(3)
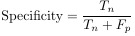

(4)
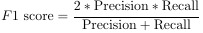

(5)
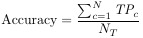

(6)
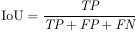

(7)
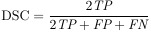

(8)
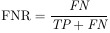

(9)
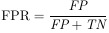

where *T_p_*/*TP* is True Positive, *F_p_*/*FP* is False Positive, *F_n_*/*FN* is False Negative, *T_n_*/*TN* is True Negative, and *N_T_* is the number of classes.

### Tumor patch classifier performance

**Figure 6.**
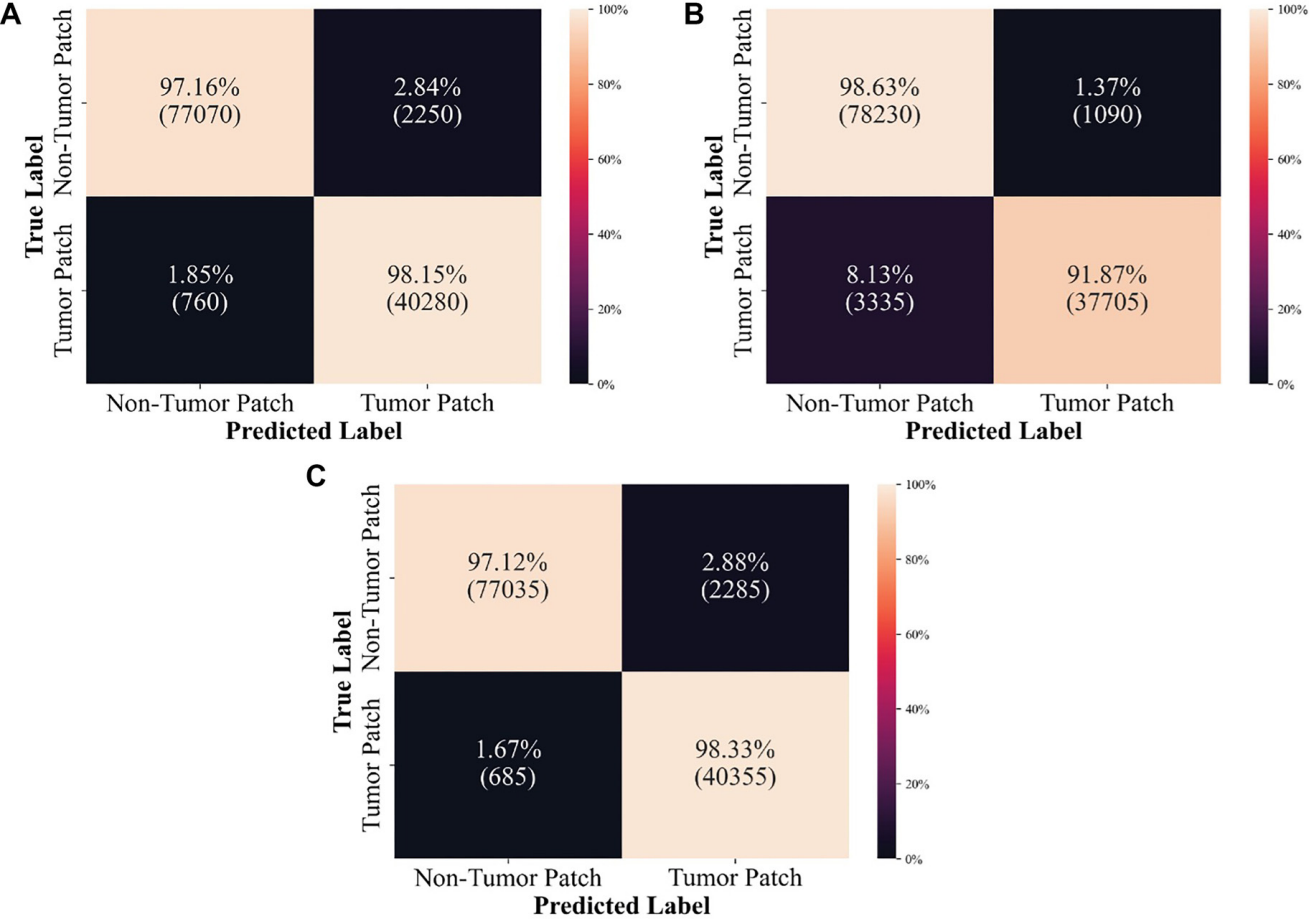
Confusion matrix of (A) EfficientNet B0; (B) Inception v3; and (C) Vision transformer model.

This section presents a performance comparison of three different deep learning architectures: EfficientNet, Inception v3, and the Vision Transformer model, in the tumor patch classification stage. EfficientNet displayed consistent performance across tumor and non-tumor classes, achieving an overall accuracy of 97.5%. For the non-tumor class, the model recorded an accuracy of 97.5%, precision of 99.02%, sensitivity of 97.16%, F1 score of 98.08%, and specificity of 98.15%. Similarly, for the tumor class, it achieved an accuracy of 97.5%, precision of 94.71%, sensitivity of 98.15%, F1 score of 96.4%, and specificity of 97.16%. The weighted average metrics were 97.5% for accuracy, 97.55% for precision, 97.5% for sensitivity, 97.51% for the F1 score, and 97.81% for specificity. Inception v3 demonstrated a slightly lower overall accuracy of 96.32% compared to EfficientNet. The non-tumor classification results showed an accuracy of 96.32%, precision of 95.91%, sensitivity of 98.63%, F1 score of 97.25%, and specificity of 91.87%. For tumor detection, the model recorded an accuracy of 96.32%, precision of 97.19%, sensitivity of 91.87%, F1 score of 94.46%, and specificity of 98.63%. The weighted average figures for Inception v3 were 96.32% for accuracy, 96.35% for precision, 96.32% for sensitivity, 96.3% for the F1 score, and 94.18% for specificity. The Vision Transformer model achieved an overall accuracy equal to EfficientNet, at 97.53%. For the non-tumor class, it recorded an accuracy of 97.53%, precision of 99.12%, sensitivity of 97.12%, F1 score of 98.11%, and specificity of 98.33%. In tumor classification, it achieved an accuracy of 97.53%, precision of 94.64%, sensitivity of 98.33%, F1 score of 96.45%, and specificity of 97.12%. The weighted averages were 97.53% for accuracy, 97.59% for precision, 97.53% for sensitivity, 97.54% for the F1 score, and 97.92% for specificity. The Vision Transformer model matched the overall accuracy of EfficientNet at 97.53% and exhibited superior sensitivity in tumor detection. This model’s performance highlights its potential for applications requiring high sensitivity to avoid missing tumor cases. The confusion matrices of the models are shown in Figure 6.

### Tumor area segmentation performance

**Figure 7.**
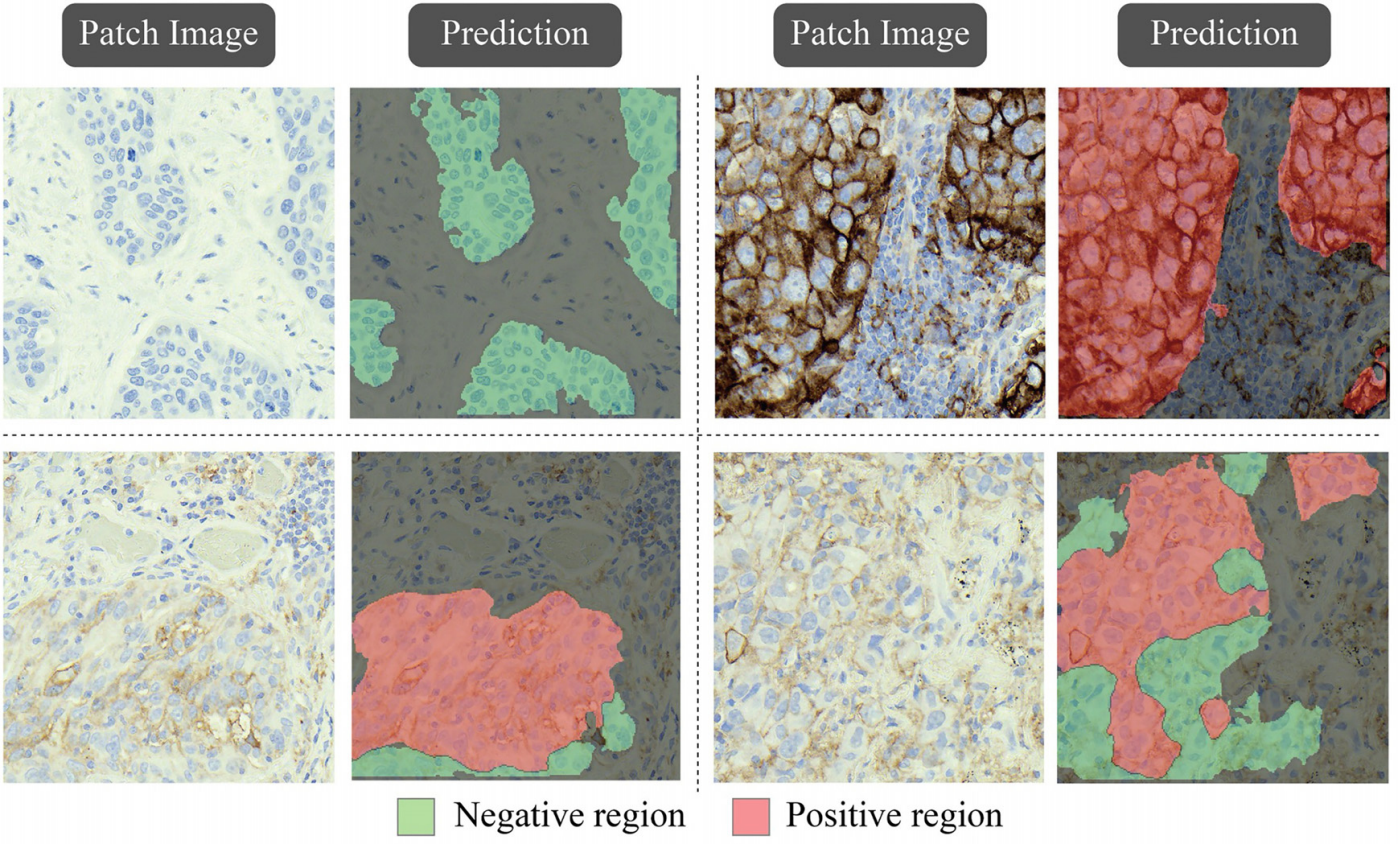
Visualization of the segmentation model performance.

A comparative evaluation of five segmentation models, including UNet, EfficientNet UNet, DenseNet UNet, DeepLabV3+, and SelfONN-based DeepLabV3+, is shown in this section. The DSC values for various models were analyzed to evaluate their segmentation performance for positive and negative tumors. UNet achieved an overall DSC of 76.28%, demonstrating consistent performance with DSC values of 76.31% for positive tumors and 76.24% for negative tumors. EfficientNet UNet significantly improved the overall DSC to 82.33%, with a remarkable 87.566% for positive tumors and 77.092% for negative tumors, indicating strong positive tumor segmentation capabilities. DenseNet UNet showed similar robustness, with an overall DSC of 81.89%, achieving 86.57% for positive tumors and 77.21% for negative tumors. DeepLabV3+ exhibited excellent performance in positive tumor segmentation with a DSC of 89.62%, though its performance in negative tumor segmentation was lower, with a DSC of 73.62%, resulting in an overall DSC of 81.62%. The SelfONN-enhanced DeepLabV3+ achieved the highest overall DSC of 83.47%, maintaining high performance in both positive (DSC of 89.58%) and negative tumors (DSC of 77.36%). These results indicate that the SelfONN enhancement particularly improves the model’s robustness, providing the most balanced and effective segmentation performance among the models evaluated. Table 2 shows the segmentation performance of the different models. Figure 7 shows segmentation predictions on patch images.

**Table 2:** Segmentation performance comparison of different architectures

**Model**	**Accuracy (%)**	**IoU (%)**	**DSC (%)**	**IoU (positive tumor) (%)**	**DSC (positive tumor) (%)**	**IoU (negative tumor) (%)**	**DSC (negative tumor) (%)**
UNet	67.97	69.07	76.28	71.12	76.31	67.02	76.24
EfficientNet UNet	70.782	76.73	82.33	84.72	87.56	68.74	77.09
DenseNet UNet	70.88	76.44	81.89	83.6	86.57	69.28	77.21
DeepLabV3+	71.58	75.27	81.62	86.89	89.62	63.66	73.62
DeepLabV3+ (SelfONN)	71.25	77.69	83.47	86.79	89.58	68.59	77.36

Abbreviations: IoU: Intersection over union; DSC: Dice similarity coefficient.

**Figure 8.**
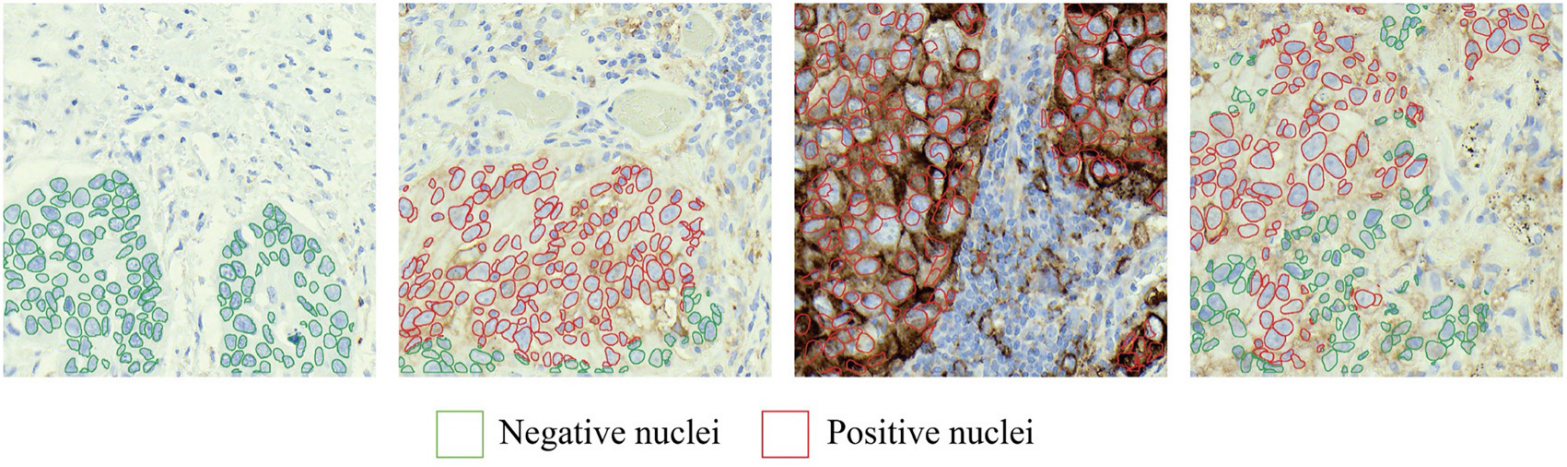
Example of cell nuclei detection using the StarDist model.

### Automated PD-L1 scoring performance

After the segmentation stage, to calculate the TPS of a WSI, the nuclei within the positive and negative areas need to be identified. The pre-trained model StarDist was used for this purpose.

Figure 8 shows examples of detected cell nuclei in patch images. The TPS of a WSI was calculated as: (10)
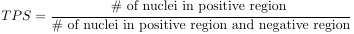


**Figure 9.**
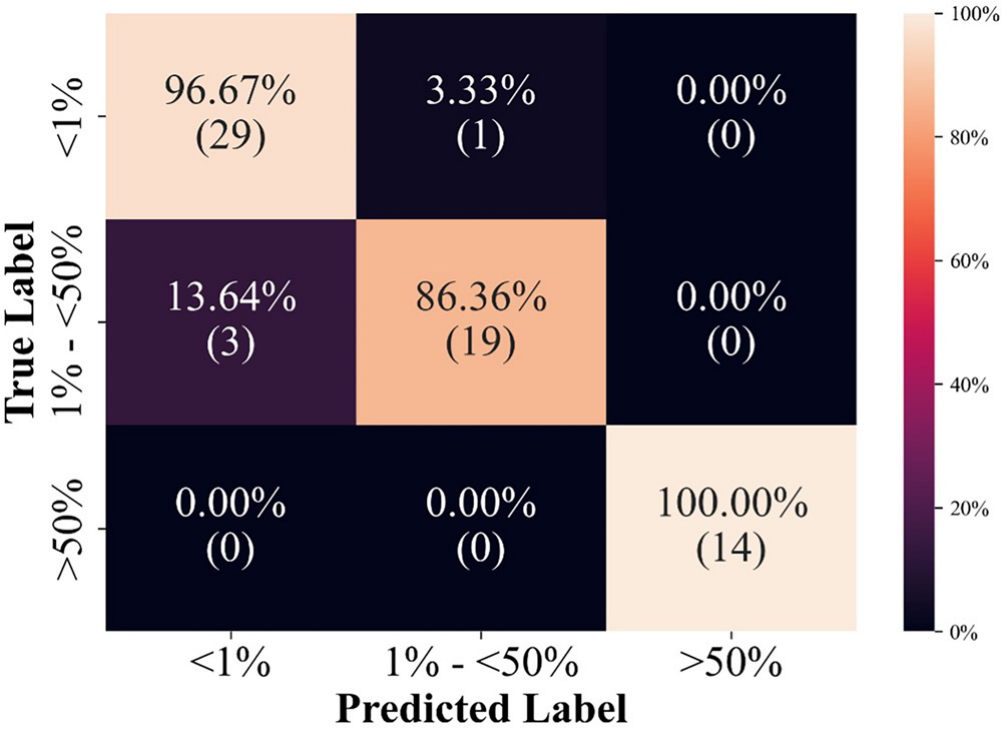
**Confusion matrix of the classification of TPS level.** Abbreviation: TPS: Tumor proportion score.

Automated PD-L1 expression was assessed based on the TPS, categorized into three levels: negative (TPS < 1%), low expression (TPS 1%–49%), and high expression (TPS ≥ 50%). According to the ground truth data provided in the dataset, there were 30 cases with negative expression, 22 with low expression, and 14 with high expression. The automated framework achieved an accuracy of 96.67% for negative cases, 86.36% for low-expression cases, and 100% for high-expression cases. An overall accuracy of 93.94% was attained, with an F1 score of 93.89%. Figure 9 shows the confusion matrix. The correlation between the ground truth TPS and the TPS predicted by the framework was 0.9635.

## Discussion

This study aimed to develop an automated framework for assessing PD-L1 expression in NSCLC using WSIs from surgical resections. The objective was to automate the evaluation of TPS to enhance clinical decision-making for ICI. Our approach comprised three key stages: tumor patch classification, tumor area segmentation, and nuclei detection.

Initially, the framework identified patches containing TCs while discarding those with artifacts or non-TCs through a classification stage. This stage demonstrated robust performance across three deep learning models: EfficientNet, Inception v3, and Vision Transformer. Both EfficientNet and Vision Transformer achieved an overall accuracy of 97.5%, surpassing Inception v3, which attained an accuracy of 96.32%. Notably, the Vision Transformer model exhibited superior sensitivity in tumor detection, underscoring its potential for applications requiring high sensitivity to avoid missing tumor cases. This may be particularly important in small biopsy samples or in cytology (e.g., cell blocks). Most existing methodologies do not employ artifact filtering, which can result in false predictions and failures in real-world scenarios. The automated framework proposed in this study processed entire slide images, accounting for artifacts and positive ICs, which should be discarded for more accurate predictions. This consideration is crucial for enhancing prediction accuracy and reliability.

The subsequent stage of the framework involved a segmentation network designed to predict positive and negative tumor regions within a patch. The segmentation performance of the models varied, with the SelfONN-enhanced DeepLabV3+ achieving the highest overall DSC of 83.47%. This model exhibited superior performance in both positive (DSC of 89.58%) and negative tumor areas (DSC of 77.36%), demonstrating its robustness and effectiveness in segmenting complex tumor regions. The incorporation of Self-ONN into the DeepLabV3+ architecture significantly enhanced performance. Previous studies primarily reported the performance of single models, particularly basic UNet models. In contrast, this work presents a comparative analysis of segmentation performance across various networks, including UNet with high-level encoders capable of capturing diverse and deeper features efficiently, as well as the modified DeepLabV3+ architecture, which represents a state-of-the-art segmentation network.

The final stage of the framework involved a deep learning network called StarDist, pre-trained to detect cell nuclei in WSIs. The number of nuclei in both positive and negative regions was determined, and the TPS was subsequently calculated for the WSI. The most common method for calculating the number of nuclei, employed by most studies, is the watershed algorithm, which relies solely on image processing. Consequently, it is susceptible to issues, such as hard stains and low-intensity cells. The StarDist model overcame these limitations and performed better across various scenarios, making it a more practical and reliable method. The automated framework’s performance in calculating the TPS from WSIs showed a strong correlation with ground truth data, achieving an overall accuracy of 93.94% and an F1 score of 93.89%. The framework performed exceptionally well in identifying high-expression cases (TPS ≥ 50%) with 100% accuracy, though it exhibited slightly lower accuracy (86.36%) in low-expression cases (TPS 1%–49%). This discrepancy highlights the ongoing challenge of accurately quantifying low PD-L1 expression levels, which is crucial for patient management and treatment planning.

Although the study achieved promising results, it has certain limitations. While it performed well in negative cases, it encountered difficulties in detecting unusually shaped TC nuclei, which are uncommon. Additionally, cases with high PD-L1 expression exhibited heterogeneous staining, leading to some inaccuracies. The study was conducted with only 66 specimens; although these were large surgical specimens, increasing the number of cases could introduce greater variability in cell morphology and stain patterns, enhancing the model’s generalizability. Moreover, while the human–machine collaborative annotation was effective in this study, more extensive and detailed human annotations could further improve model performance due to the subjective nature of the problem.

Scopes of future work can focus on further refining the segmentation and classification models, particularly on improving the accuracy of low-expression PD-L1 cases. Additionally, including a more diverse range of NSCLC subtypes to expand the dataset and PD-L1 staining patterns will be essential for generalizing the model’s applicability. The incorporation of multimodal data, such as genomic and clinical information, could also enhance the framework’s predictive power.

## Conclusion

The automated framework developed in this study shows significant promise in the field of digital pathology, offering a valuable tool for the accurate and efficient assessment of PD-L1 expression in NSCLC. By harnessing the power of deep learning, this framework provides a reliable and scalable method for automating PD-L1 TPS evaluation, a critical factor in determining eligibility for ICI. This study developed a robust deep learning-based model that achieved high accuracy across several essential tasks, including tumor patch classification, segmentation, and cancer cell nuclei detection. The framework demonstrated impressive performance in distinguishing between tumor and non-tumor regions and accurately identifying PD-L1-positive cancer cell nuclei, a key component in TPS calculation. The ability of this automated system to consistently and accurately quantify PD-L1 expression highlights its potential as a powerful tool for pathologists, helping to reduce the subjectivity and variability that often arise in manual assessments. The findings of this research underscore the broader potential of AI-driven solutions in improving diagnostic accuracy, streamlining workflows in pathology labs, and facilitating more precise patient selection for treatment with ICI. By integrating such automated systems into clinical practice, there is a significant opportunity to enhance both the speed and consistency of cancer diagnosis and treatment decisions. As the healthcare sector continues to adopt AI-based technologies, this framework could play a critical role in optimizing personalized treatment strategies, improving patient outcomes, and potentially reducing the overall cost and burden of cancer care. Future work could further refine the model, extend its applicability to other cancer types, and integrate it into routine clinical workflows, reinforcing the growing role of AI in advancing precision medicine.

## Data Availability

The processed dataset used in this study can be made available upon a reasonable request to the corresponding author.
